# Activation of Nrf2 by miR-152 Inhibits Doxorubicin-Induced Cardiotoxicity via Attenuation of Oxidative Stress, Inflammation, and Apoptosis

**DOI:** 10.1155/2021/8860883

**Published:** 2021-01-26

**Authors:** Wen-Bin Zhang, Xin Lai, Xu-Feng Guo

**Affiliations:** ^1^Department of Oncology, Renmin Hospital of Wuhan University, Wuhan 430060, China; ^2^Department of Cardiology, Renmin Hospital of Wuhan University, Wuhan 430060, China

## Abstract

Doxorubicin (DOX) could trigger congestive heart failure, which largely limited the clinical use of DOX. microRNAs (miRNAs) were closely involved in the pathogenesis of DOX-induced cardiomyopathy. Here, we aimed to investigate the effect of miR-152 on DOX-induced cardiotoxicity in mice. To study this, we used an adeno-associated viral vector to overexpress miR-152 in mice 6 weeks before DOX treatment, using a dose mimicking the concentrations used in the clinics. In response to DOX injection, miR-152 was significantly decreased in murine hearts and cardiomyocytes. After DOX treatment, mice with miR-152 overexpression in the hearts developed less cardiac dysfunction, oxidative stress, inflammation, and myocardial apoptosis. Furthermore, we found that miR-152 overexpression attenuated DOX-related oxidative stress, inflammation, and cell loss in cardiomyocytes, whereas miR-152 knockdown resulted in oxidative stress, inflammation, and cell loss in cardiomyocytes. Mechanistically, this effect of miR-152 was dependent on the activation of nuclear factor (erythroid-derived 2)-like 2 (Nrf2) in response to DOX. Notably, Nrf2 deficiency blocked the protective effects of miR-152 against DOX-related cardiac injury in mice. In conclusion, miR-152 protected against DOX-induced cardiotoxicity via the activation of the Nrf2 signaling pathway. These results suggest that miR-152 may be a promising therapeutic target for the treatment of DOX-induced cardiotoxicity.

## 1. Introduction

Doxorubicin (DOX), a quinone-containing anthracycline, is effective in the treatment of severe leukemia and malignant lymphomas [1]. Cardiomyocyte loss and congestive heart failure, however, compromised the clinical use of DOX [2]. The mechanism of DOX-induced cardiotoxicity was complex, but accumulating evidence indicated that oxidative stress and inflammation were closely involved [3, 4].

Available laboratory evidence showed that reactive oxygen species (ROS) and subsequent lipid peroxidation could be detected in the hearts within three hours after DOX treatment [5–8]. In addition, nuclear factor kappa-B (NF-*κ*B) activation by DOX was observed very early in the hearts of mice [9]. Accumulation of ROS and inflammation caused caspase-3 activation and resulted in myocardial apoptosis [10]. Therefore, inhibition of these alterations would be of great significance to the treatment of DOX-related cardiac toxicity.

Nuclear factor (erythroid-derived 2)-like 2 (Nrf2) encodes multiple antioxidant genes and detoxifying enzymes [11]. Under physiological conditions, Nrf2 binds Kelch-like ECH-associated protein 1 (Keap1) and Cul3 ubiquitin ligase. Upon stimuli, Nrf2 is released from Keap1 and binds the antioxidant responsive element, leading to the transcription of Nrf2-dependent antioxidant genes [11]. Decreased Nrf2 expression was observed in DOX-treated hearts, and restoration of Nrf2 expression by a pharmacological agent could attenuate DOX-induced cardiotoxicity in mice [12]. Therefore, we speculated that activation of Nrf2 might largely rescue DOX-triggered cardiotoxicity.

microRNAs (miRNAs) bind to the 3′-untranslated region (3′-UTR) of the targeted genes and cause degradation of protein expression [13]. miRNAs have been reported to regulate the activation of the Keap1-Nrf2 pathway [14, 15]. miR-200a could target Keap1 mRNA and promote degradation of Keap1, resulting in Nrf2 activation to protect against DOX-induced cardiotoxicity in mice [16]. The miR-152 family has been implicated in processes of immunomodulation, cell growth, and proliferation [17]. miR-152 overexpression improved cardiomyocyte viability and prevented hypoxia-induced cell injury in vitro [18]. In addition, miR-152 upregulation suppressed angiotensin II-induced cardiomyocyte apoptosis [19]. A previous study found that miR-152 protected against neuron injury through upregulation of the Nrf2 signaling pathway by targeting postsynaptic density protein-93 [20]. These observations raised the possibility that miR-152 would protect against DOX-triggered cardiotoxicity via regulation of Nrf2. In this study, we found that miR-152 targeted Keap1 to activate Nrf2 and protected mice from DOX-induced cardiotoxicity.

## 2. Materials and Methods

### 2.1. Antibodies and Reagents

Antibodies against NF-*κ*B (ab16502), Lamin B (ab16048), Nrf2 (ab62352), Bax (ab32503), caspase3 (ab13847), cleaved-caspase3 (ab2302), Keap1 (ab119403) and GAPDH (ab181602) were provided by Abcam (Cambridge, UK). Mouse 3-nitrotyrosine (3-NT) ELISA kit (ab116691) and 4-hydroxynonenal (4-HNE)-protein adducts kit (ab238538) were acquired from Abcam. DOX (#D1515) and 2′,7′-dichlorodihydrofluorescein diacetate (DCFH-DA) were provided by Sigma. CardioTACS In Situ Apoptosis Detection Kit was obtained from Millipore (Billerica, MA, USA). Malondialdehyde (MDA, S0131), glutathione measurement kits (S0053), and total superoxide dismutase activity (SOD, S0101) were obtained from Beyotime Institute of Biotechnology (Shanghai, China).

### 2.2. Animals and Treatment

The protocols involving the use of animals were approved by the Institutional Animal Research Committee of Renmin Hospital of Wuhan University. An adeno-associated virus 9 (AAV9) system carrying miR-152 or miR-scramble under the cTnT promoter was generated by Genecopia (Shanghai, China). To overexpress miR-152 in the hearts, mice (male, age: 9-11 weeks) were given 5 × 10^11^ viral genome of AAV9-miR-152 or AAV9-miR-scramble by a single tail vein injection as described [21, 22]. These mice were injected with a single dose of DOX (15 mg/kg) at 6 weeks after AAV9 infection. We used normal saline (NS) to dissolve DOX. The animals were observed for 5 days after DOX injection; after that, animals were sacrificed. Cardiac Nrf2 depletion was achieved by a tail vein injection of AAV9 carrying Nrf2 small hairpin RNAs (shNrf2) or scrambled shRNA (5 × 10^11^ viral genome/mouse) under the cTnT promoter (Genecopia).

### 2.3. Hemodynamics

After adequate anesthetization with 1.5% isoflurane, the hearts were excised and a pressure-volume catheter (Millar Instruments, USA) was inserted through an apical stab into the ventricle to measure cardiac function. Hemodynamic measurements were analyzed using an IOX software (EMKAtech).

### 2.4. RNA Extraction and Real-Time RT-PCR

Total RNA was extracted from the heart samples or cultured cardiomyocytes. Reverse transcriptional reactions were performed using the PrimeScript RT Reagent Kit (#RR036B, TaKaRa, Otsu, Japan). SYBR Green PCR Master Mix (04887352001, Roche) was used to detect the PCR amplification products. The mRNA levels of the target genes were normalized to those of GAPDH. The levels of miR-152 were detected using an All-in-One™ miRNA qPCR Kit (Genecopia).

### 2.5. Western Blot Analysis

Heart samples or cultured cardiomyocytes were lysed with a RIPA lysis buffer. An equal amount of proteins were separated in 10% SDS-PAGE gels and then transferred onto the PVDF membranes (Millipore, CA, USA). The membranes were incubated with the primary antibodies (dilution, 1 : 1000) overnight at 4°C. After that, these bands were incubated with appropriate horseradish peroxidase-conjugated secondary antibodies and visualized using enhanced chemiluminescent reagents (Promega, Madison, USA). Signals were quantified by the ImageJ software (Bio-Rad, Hercules, USA). GAPDH was used as the internal control. CelLytic™ NuCLEAR™ Extraction Kit (NXTRACT-1KT, Sigma) was used to extract the nuclear protein in our study.

### 2.6. NF-*κ*B and Nrf2 Activation Assay

Transcription factor activation in heart samples and cell extracts were detected using TransAM® NF*κ*B Activation Assay Kits and TransAM® Nrf2 Activation Assay Kits. The two kits are DNA-binding ELISAs that simplify the study of transcription factor activation, which were obtained from Active Motif.

### 2.7. Biochemical Detection

Fresh heart samples or cultured cardiomyocytes were homogenized in cold saline, and the supernatant fraction was collected to detect myocardial tumor necrosis factor-*α* (TNF-*α*), interleukin-6 (IL-6), IL-10, and interferon-*γ* (IFN-*γ*). TNF-*α* Mouse ELISA Kit (#BMS607-3), IL-6 Mouse ELISA Kit (#BMS603HS), and IL-10 Mouse Instant ELISA™ Kit (#BMS614INST) were provided by Invitrogen. IFN-*γ* (mouse) ELISA Kit, provided by Biovision (Shanghai, China), was used to detect myocardial IFN-*γ*. To evaluate oxidative damage, reduced glutathione (GSH), and oxidized GSH (GSSG), MDA, 4-HNE, and 3-NT were detected according to standard procedures.

To reflect cardiac injury after DOX treatment, lactate dehydrogenase (LDH), creatine kinase (CK), and cardiac troponin I (cTnI) were detected according to standard procedures. LDH Cytotoxicity Assay Kit (C0016) was obtained from Beyotime Institute of Biotechnology, and Creatine kinase assay kit (A032-1-1) was provided by Nanjing Jiancheng Bioengineering Institute (Nanjing, China). cTnI assay kit was obtained from Life Diagnostics, Inc. (West Chester, PA).

### 2.8. Apoptosis Assay

Heart paraffin sections were stained with terminal deoxynucleotidyl transferase-mediated nick-end labelling (TUNEL) staining to assess myocardial apoptosis. We also detected myocardial caspase 3 activity using the Caspase 3 Activity Assay Kit (C1116, Beyotime).

### 2.9. Cell and Treatment

Neonatal rat cardiac myocytes (NRCMs) were isolated according to a previous method [23, 24]. These cells were assigned into four groups: PBS+ miR-scramble, PBS+miR-152, DOX+miR-scramble, and DOX+miR-152 groups. The cardiomyocytes were pretreated with micrON miR-152 (50 nmol/l) or scramble control for 48 hours and then incubated with DOX at 5 *μ*g/ml or the same volume of PBS for 24 h. micrON miR-152 and scramble control were generated by Ribobio Technology (Guangzhou, China). To knock down miR-152 in NRCMs, these cells were subjected to a miR-152 inhibitor (50 nmol/l, Ribobio Technology) for 48 hours. To verify the hypothesis that the inflammatory response was secondary to the ROS production after miR-152 inhibition, NRCMs were pretreated with a nonspecific ROS inhibitor (*N*-acetyl-L-cysteine, NAC, 5 mmol/l) for 12 hours. To verify the hypothesis that miR-152 exerted protection via activation of Nrf2, cells were treated with siNrf2 (50 nmol/l) to knock down Nrf2. siNrf2 was generated by Invitrogen, and scrambled siRNA was used as a nonspecific control. Cell viability was determined by the cell counting assay (CCK-8; Dojindo Molecular Technologies, Rockville, USA) according to the manufacturer's instruction.

### 2.10. Intracellular ROS and Superoxide Detection

The levels of intracellular ROS were determined by spectrophotometry using DCFH-DA staining [25]. NRCMs were reacted with DCFH-DA (10 *μ*mol/l) for 30 min at 37°C in the dark. And then, the cells were washed three times with PBS, and the fluorescence intensity was determined using a microplate reader (Biotek Instruments, VT, USA). We used Superoxide Anion Assay Kit (#CS1000, Sigma) to detect superoxide levels according to standard procedures.

### 2.11. Mitochondrial Respiration Complex I Activity and ATP Level Detection

Myocardial mitochondrial respiration complex I in the hearts was detected with a MitoCheck Complex I Activity Assay Kit. The ATP concentration was measured by using an ATP assay kit (Beyotime, Shanghai) according to the manufacturer's protocol.

### 2.12. Luciferase Assay

Keap1 3′-UTR reporter plasmid (containing the miR-152-binding sites) was generated by Genecopia, which was then transfected to NRCMs. The cardiomyocytes were treated with micrON miR-152 (50 nmol/l) or scramble control for 48 hours. Afterwards, Keap1 3′-UTR luciferase activity was tested via a Promega kit.

### 2.13. Statistical Analysis

All data are shown as means ± SEM. The two-tailed unpaired *T*-test was carried out to examine the significance between the two groups. Comparisons between multiple groups were performed using one-way ANOVA followed by a post hoc Bonferroni post hoc analysis for data meeting homogeneity of variance requirements. Statistical significance was accepted at a value of *P* < 0.05.

## 3. Results

### 3.1. miR-152 Level Was Downregulated in DOX-Treated Hearts and Cardiomyocytes

**Figure 1:**
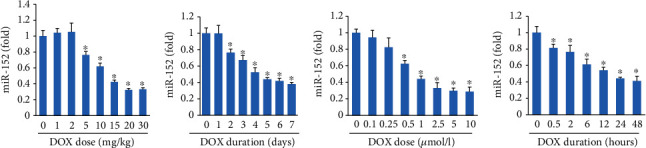
miR-152 expression after DOX treatment. (a, b) The miR-152 expression after DOX injection in mice (*n* = 6). (c, d) The miR-152 expression after DOX injection in neonatal rat cardiomyocytes (*n* = 6). ^∗^*P* < 0.05 compared with NS (PBS) group. Values represent the mean ± SEM.

To investigate the involvement of miR-152 in the progression of DOX-related cardiac injury, we examined its levels in the hearts of DOX-treated mice. The cardiac miR-152 levels were significantly decreased in dose- and time-dependent manners after DOX exposure (Figures 1(a) and 1(b)). Using NRCMs, we also found that DOX treatment dose- and time-dependently decreased the expression of miR-152 in the cells (Figures 1(c) and 1(d)).

### 3.2. Overexpression of miR-152 Attenuated DOX-Induced Cardiac Injury and Improved Cardiac Function

**Figure 2:**
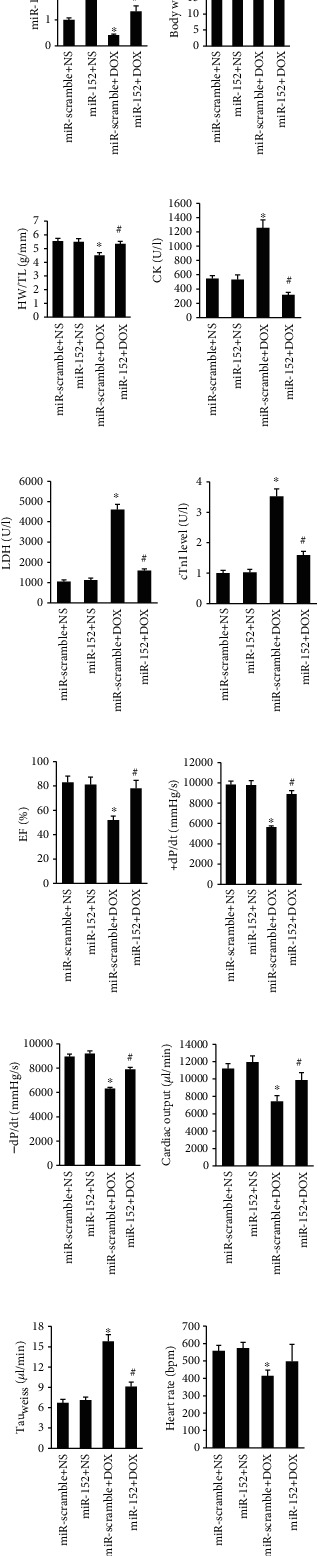
miR-152 overexpression improved cardiac function in mice with DOX treatment. (a) The miR-152 expression after AAV injection (*n* = 6). (b) Body weight of four groups (*n* = 12‐13). (c) Statistical results of the heart weight (HW)/tibia length (TL) (*n* = 12‐13). (d–f) The level of CK, LDH, and cTnI in DOX-treated mice (*n* = 6). (g–i) The alteration in EF, +dP/dt, and -dP/dt in mice (*n* = 8). (j, k) Cardiac output and Tau_Weiss_ in mice (*n* = 8). (l) Heart rate (*n* = 8). Values represent the mean ± SEM. ^∗^*P* < 0.05 versus NS+miR-scramble; ^#^*P* < 0.05 versus DOX+miR-scramble.

To address the function of miR-152 in DOX-related cardia injury in vivo, mice were given 5 × 10^11^ viral genome of AAV9-miR-152 or AAV9-miR-scramble by a tail vein injection to overexpress miR-152 in the hearts. As indicated by Figure 2(a), the decreased myocardial miR-152 expression caused by DOX was successfully restored in the hearts of mice with AAV9-miR-152 injection (Figure 2(a)). After 5 days of DOX injection, mice with miR-152 expression exhibited higher body weights and heart-to-tibal length ratios than those of mice with AAV9-miR-scramble injection (Figures 2(b) and 2(c)). Furthermore, DOX-treated miR-152-overexpressed mice exhibited alleviated cardiac injury, as evidenced by the decreased plasma CK, LDH, and cTnI (Figures 2(d)–2(f)). DOX injection resulted in decreased ejection fraction (EF), maximum first derivative of ventricular pressure with respect to time (+dP/dt), -dP/dt, and cardiac output. However, these pathological alterations were attenuated after miR-152 overexpression in mice (Figures 2(g)–2(j)). miR-152 also decreased the elevation of Tau_Weiss_ in DOX-treated mice (Figure 2(k)). We also detected heart rate and found that miR-152 cannot affect the decreased heart rate in response to DOX (Figure 2(l)).

### 3.3. miR-152 Attenuated Myocardial Inflammation and Oxidative Damage in DOX-Treated Mice

**Figure 3:**
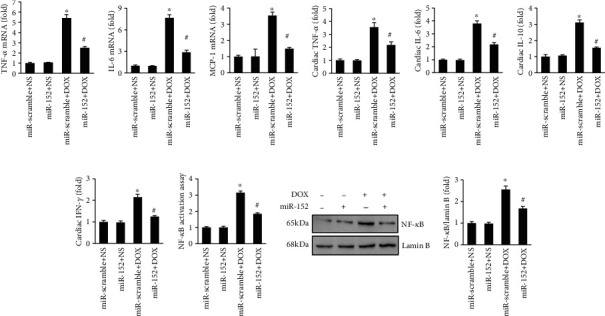
miR-152 supplementation attenuated myocardial inflammation accumulation in mice with DOX. (a–c) The mRNA levels of inflammatory factors in the hearts (*n* = 6). (d–g) Myocardial inflammatory factors as detected by ELISA (*n* = 6). (h) NF-*κ*B activity in mice (*n* = 6). (i) NF-*κ*B in the nucleus (*n* = 6). Values represent the mean ± SEM. ^∗^*P* < 0.05 versus NS+miR-scramble, ^#^*P* < 0.05 versus DOX+miR-scramble.

In response to DOX, myocardial TNF-*α*, IL-6, and MCP-1 mRNA levels were significantly increased (Figures 3(a)–3(c)). However, these pathological elevations were decreased after miR-152 injection in mice (Figures 3(a)–3(c)). As indicated in Figures 3(d)–3(f), the overexpression of miR-152 significantly decreased myocardial cytokine protein levels, with the reduction found in TNF-*α*, IL-6, IL-10, and IFN-*γ* (Figures 3(d)–3(g)). Next, we detected the DNA binding of NF-*κ*B and found that miR-152 significantly decreased the elevated NF-*κ*B activation in DOX-treated hearts (Figure 3(h)). DOX-induced nuclear translocation of NF-*κ*B was also inhibited by miR-152 supplementation (Figure 3(i)).

**Figure 4:**
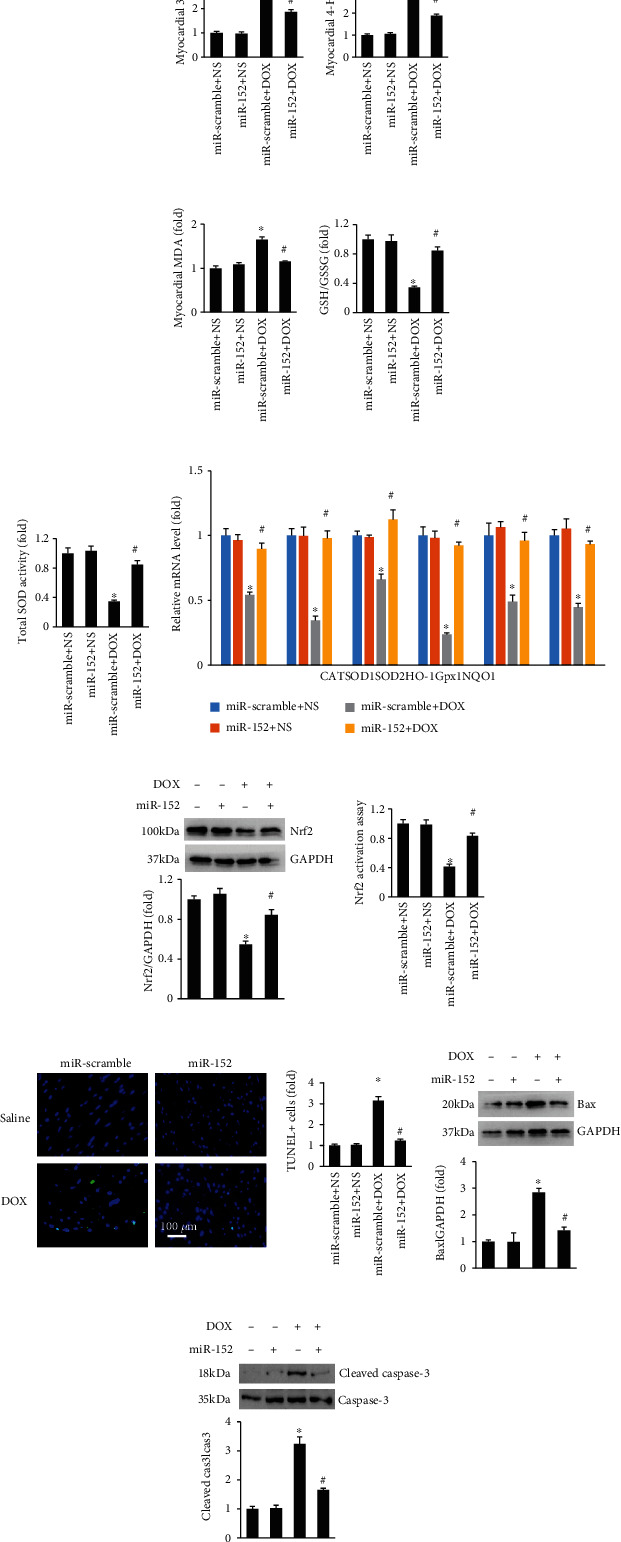
miR-152 reduced oxidative stress in DOX-treated mice. (a, b) The level of myocardial 3-NT and 4-HNE in the hearts (*n* = 6). (c, d) The levels of cardiac MDA and GSH/GSSG (*n* = 6). (e) Total SOD activity (*n* = 6). (f) The levels of Nrf2-regulated genes in the hearts (*n* = 6). (g) Protein expression of nuclear Nrf2 (*n* = 6). (h) Nrf2 activity assay (*n* = 6). (i) TUNEL staining (*n* = 6). (j, k) The protein expression of Bax and cleaved-caspase3 in the hearts (*n* = 6). Values represent the mean ± SEM. ^∗^*P* < 0.05 versus NS+miR-scramble, ^#^*P* < 0.05 versus DOX+miR-scramble.

Next, we detected myocardial oxidative stress in mice. Elevated 3-NT, 4-HNE, and MDA in response to DOX were suppressed by miR-152 in mice (Figures 4(a)–4(c)). DOX injection decreased the ratio of GSH to GSSG and total SOD activity, and those downregulations were blocked by miR-152 (Figures 4(d) and 4(e)). Further detection revealed that the decreased mRNA levels of catalase (CAT), SOD1, SOD2, heme oxygenase 1 (HO-1), glutathione peroxidase 1 (Gpx1), and NAD(P)H:quinone oxidoreductase 1 (NQO1) were restored by miR-152 in DOX-treated mice (Figure 4(f)). The decreased Nrf2 protein expression in response to DOX treatment was also restored by miR-152 (Figure 4(g)). miR-152 significantly increased the decreased Nrf2 activation in DOX-treated hearts (Figure 4(h)). We next evaluated mitochondrial respiration complex I activity and ATP levels. Complex I activity and ATP levels were significantly reduced in the mitochondria of DOX mice compared with control mice and which were significantly increased by miR-152 (Figure S1A-B). In addition, miR-152 significantly decreased the percentage of TUNEL-positive nuclei in DOX-treated hearts (Figure 4(i)). Western analysis revealed that miR-152 decreased the protein expression of Bax and cleaved-caspase3 in DOX-treated hearts (Figures 4(j) and 4(k)).

### 3.4. miR-152 Attenuated DOX-Related Cardiomyocyte Injury In Vitro

**Figure 5:**
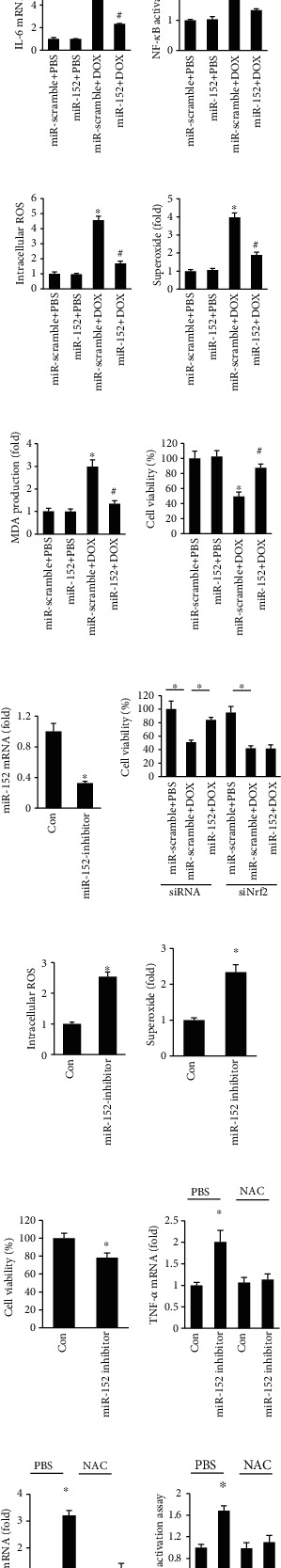
The role of miR-152 in cardiomyocytes. (a) miR-152 expression in cardiomyocytes (*n* = 6). (b, c) The mRNA levels of inflammatory factors in cardiomyocytes (*n* = 6). (d) NF-*κ*B activity in cardiomyocytes (*n* = 6). (e, f) ROS and superoxide in cardiomyocytes (*n* = 6). (g) MDA production in cardiomyocytes (*n* = 6). (h) Cell viability in cardiomyocytes (*n* = 6). (i) miR-152 expression in cardiomyocytes (*n* = 6). (j) The mRNA levels of inflammatory factors in cardiomyocytes (*n* = 6). (k, l) ROS and superoxide in cardiomyocytes (*n* = 6). (m) Cell viability in cardiomyocytes (*n* = 6). (n, o) The mRNA levels of inflammatory factors in cardiomyocytes (*n* = 6). (p) NF-*κ*B activity in cardiomyocytes (*n* = 6). Values represent the mean ± SEM. ^∗^*P* < 0.05 versus the matched control.

To explore the effects of miR-152 in vitro, we infected NRCMs with micrON miR-152 or scramble control. DOX-induced cardiomyocyte inflammation accumulation and NF-*κ*B activation in the miR-152 overexpression group were markedly ameliorated compared to those in the control group (Figures 5(a)–5(d)). miR-152 supplementation largely reduced the production of ROS, superoxide, and MDA in DOX-treated cells (Figures 5(e)–5(g)). The decreased cardiomyocyte viability was improved after miR-152 overexpression (Figure 5(h)). In contrast, miR-152 inhibition in NRCMs resulted in inflammation accumulation, ROS, and superoxide production, thus impairing cell viability even without any stimuli (Figures 5(i)–5(m)). To verify the hypothesis that the inflammatory response was secondary to the production of ROS after miR-152 inhibition, NAC was used. NAC pretreatment abolished the accumulation of inflammation and NF-*κ*B activation caused by miR-152 inhibition (Figures 5(n)–5(p)).

### 3.5. miR-152 Provided Cardiac Protection via Activation of Nrf2

**Figure 6:**
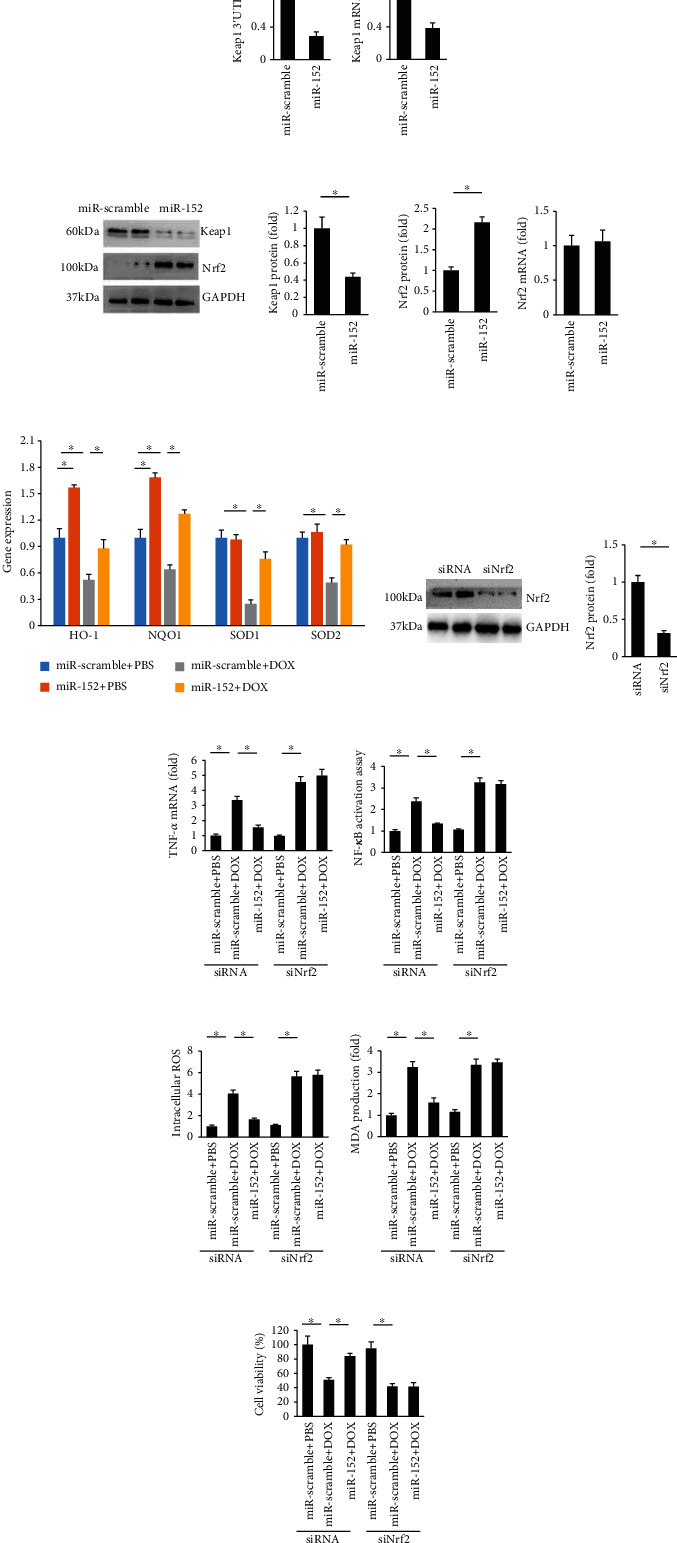
miR-152 targeted Keap1 and activated Nrf2. (a) Luciferase assay (*n* = 6). (b) Keap1 mRNA level (*n* = 6). (c) The protein expression of Nrf2 and Keap1 (*n* = 6). (d) Nrf2 mRNA level (*n* = 6). (e) The levels of Nrf2-regulated genes (*n* = 6). (f) The protein expression of Nrf2 (*n* = 6). (g, h) TNF-*α* mRNA level and NF-*κ*B activity in cardiomyocytes (*n* = 6). (i, j) ROS and MDA production (*n* = 6). (k) Cell viability (*n* = 6). Values represent the mean ± SEM. ^∗^*P* < 0.05 versus the matched control.

The data in our study found that miR-152 could target the 3′-UTR of Keap1, and decreased Keap1 mRNA in cells with miR-152 overexpression (Figures 6(a) and 6(b)). Significantly, Keap1 protein expression was downregulated, and Nrf2 expression was upregulated in miR-152-overexpressed cells (Figure 6(c)). We also detected the mRNA levels of Nrf2 and found that miR-152 did not affect the mRNA levels of Nrf2 in cardiomyocytes (Figure 6(d)). Furthermore, mRNA expression of several key Nrf2-dependent genes, including HO1, NQO1, SOD1, and SOD2, was significantly restored in miR-152-overexpressing cells (Figure 6(e)). Next, we tested whether Nrf2 activation is essential for miR-152-induced cardiomyocyte protection against DOX-related cardiac injury. Cells were treated with siNrf2 to achieve Nrf2 knockdown, and Nrf2 depletion was confirmed by Western blotting (Figure 6(f)). As expected, miR-152 lost its protection in cardiomyocytes against DOX-related injury, as reflected by the TNF-*α* mRNA, NF-*κ*B activation, ROS production, MDA content, and cell viability (Figures 6(g)–6(k)).

**Figure 7:**
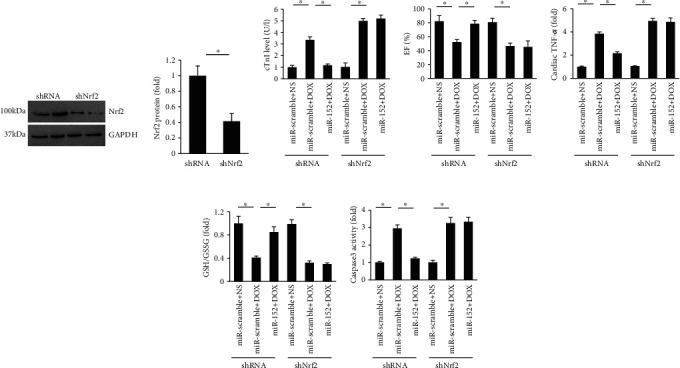
miR-152 lost protection in Nrf2-deficient mice. (a) Nrf2 expression (*n* = 6). (b) The level of plasma cTnI (*n* = 8). (c) EF in DOX-treated mice (*n* = 8). (d) Cardiac TNF-*α* level (*n* = 6). (e) GSH/GSSG ratio (*n* = 6). (f) The activity of caspase3 (*n* = 6). Values represent the mean ± SEM. ^∗^*P* < 0.05 versus the matched control.

To further verify the hypothesis that miR-152 exerted its protection via activation of Nrf2 in mice, we knocked down myocardial Nrf2 in mice and found that there were no difference in plasma cTnI, EF, cardiac TNF-*α* level, GSH/GSSG, and caspase3 activity between miR-scramble+DOX+shNrf2 and miR-152+DOX+shNrf2 groups (Figures 7(a)–7(f)).

## 4. Discussion

The expression and potential function of miR-152 in DOX-related cardiac injury were unknown. The study by Zhang et al. showed that miR-152 was closely involved in the antiapoptotic effect of tanshinone IIA in cardiomyocytes [19]. miR-152 promoted the regeneration of the damaged neonatal heart through glycolysis [26]. However, there sounded quite a different voice that the upregulation of miR-152 expression in the failing myocardium contributed to heart failure pathophysiology [27]. Here, for the first time, we demonstrated that DOX decreased miR-152 expression, and supplementation of miR-152 resulted in cardioprotection from DOX-induced cardiotoxicity in mice and cardiomyocytes. More specifically, our data illustrated that miR-152 had the capacity to attenuate myocardial inflammation and oxidative damage and cardiomyocyte apoptosis, thus preventing cardiac dysfunction in mice. These findings positively suggest miR-152 supplementation may be a helpful approach to prevent DOX-related cardiac injury.

DOX-induced cardiomyopathy occurred primarily via the generation of ROS in the cardiomyocyte [28]. This mechanism separated DOX-induced cardiomyopathy from its antineoplastic activity. DOX could react with the hydrogen peroxide to yield hydroxyl radical, thus reacting with protein and lipids and thereby causing cell damage [29]. Here, we also found that DOX increased ROS, superoxide, and MDA production and decreased antioxidant activities in mice and cardiomyocytes. However, these pathological alterations were largely attenuated by miR-152 treatment, implying that the protection of miR-152 might be partly mediated by the amelioration of oxidative damage. In addition, NF-*κ*B activation by DOX was observed in the hearts of mice, and DOX also could induce myocardial inflammation [4, 9]. Consistently, cardiac NF-*κ*B activation and mRNA levels of inflammatory factors were found to be increased upon DOX treatment, but prevented by miR-152 supplementation, accompanied by an amelioration of cardiac dysfunction. These findings were consistent with a previous study [30]. Using NAC, we found that ROS depletion abolished the accumulation of inflammation and NF-*κ*B activation caused by miR-152 inhibition, suggesting that the inflammatory response was secondary to the production of ROS in miR-152-deficient cardiomyocytes, which was also in line with a recent study found that inflammatory response was not the main biological factor and secondary to ROS production during acute DOX injury [31]. DOX-induced accumulation of ROS resulted in cytochrome c release followed by caspase-3 activation and cell apoptosis [10]. In the present study, we observed a significant elevation in Bax and cleaved-caspase 3 expression after DOX injection compared with the control groups. Conversely, we found that miR-152 decreased the percentage of TUNEL-positive nuclei and the protein expression of Bax and cleaved-caspase 3 expression in DOX-treated hearts. These findings suggested that the improvement of cardiac function after miR-152 supplementation was partly dependent on the attenuation of myocardial inflammation, oxidative damage, and apoptosis.

Nrf2 was closely involved in the development of DOX-related oxidative damage and myocardial apoptosis. Decreased Nrf2 expression and activity were observed in DOX-induced acute cardiac injury [22], and restoration of Nrf2 by a pharmacological agent attenuated DOX-induced cardiotoxicity [12]. Recent studies demonstrate that miRNA-induced silencing of Keap1 is a novel strategy to regulate Nrf2 activation. miR-7 could target Keap1 and activate Nrf2 signaling in differentiated human neural progenitor cells [32]. miR-141 activated Nrf2 signaling by targeting and silencing Keap1 to protect cells against ultraviolet-induced oxidative stress [33]. It has been reported that miR-152 protected against neuron injury through the upregulation of Nrf2 signaling pathway by targeting postsynaptic density protein-93 [20]. The results of this study suggested that miR-152 was a direct and specific Keap1-targeting miRNA, which could regulate the Keap1-Nrf2 cascade in the hearts. In cardiomyocytes, forced miR-152 overexpression suppressed Keap1 3′-UTR reporter luciferase activity and downregulated Keap1 mRNA and protein expression, thus resulting in Nrf2 activation. To verify the hypothesis that miR-152 exerted cardiac protection via activation of Nrf2, we depleted Nrf2 expression in the hearts and found that miR-152 lost cardiac protection against DOX-related cardiac injury in Nrf2-deficient hearts, suggesting that the protection provided by miR-152 was dependent on the activation of Nrf2. Therefore, miR-152 overexpression provides a novel strategy to protect the hearts from DOX-related cardiac injuries.

In this study, we did not elevate whether miR-152 supplementation would promote tumor growth. We also did not elevate whether miR-152 supplementation compromises therapeutic DOX levels. These are the limitations of our study.

In conclusion, we discovered that miR-152 is a novel activator of Nrf2 in the setting of DOX-related cardiac injury. Cardiac miR-152 silenced Keap1 and activated Nrf2, leading to the attenuation of myocardial inflammation, oxidative stress, and apoptosis. This signaling cascade offers a potentially novel therapeutic target for the treatment of DOX-related cardiac injury.

## Data Availability

The data that support the findings of this study are available from the corresponding author upon reasonable request.

## Supplementary Materials

Supplementary Materials Figure S1 The alterations in mitochondrial respiration complex I activity and ATP levels. (A) Mitochondrial respiration complex I activity (*n* = 6). (B) ATP levels (*n* = 6). Values represent the mean ± SEM. ^∗^*P* < 0.05  versus NS+miR-scramble, ^#^*P* < 0.05 versus DOX+miR-scramble.
