# Supplementary material for: Activation of Nrf2 by miR-152 Inhibits Doxorubicin-Induced Cardiotoxicity via Attenuation of Oxidative Stress, Inflammation, and Apoptosis
Source: Oxid Med Cell Longev. 2021 Jan 26;2021:8860883. doi: 10.1155/2021/8860883 (PMC7857911; doi:10.1155/2021/8860883)
Supplement: Supplementary Materials — Figure S1 The alterations in mitochondrial respiration complex I activity and ATP levels. (A) Mitochondrial respiration complex I activity (n = 6). (B) ATP levels (n = 6). Values represent the mean ± SEM. ∗P < 0.05 versus NS+miR-scramble, #P < 0.05 versus DOX+miR-scramble. [file 8860883.f1.docx]

**Supplement**

**Activation of Nrf2 by miR-152 inhibits doxorubicin-induced cardiotoxicity via attenuation of oxidative stress, inflammation and apoptosis**

Wen-Bin Zhang^1^, Xin Lai^2^, Xu-Feng Guo^1^

^1^Department of Cancer Center, Renmin Hospital of Wuhan University, Wuhan, 430060, China

^2^Department of Cardiology, Renmin Hospital of Wuhan University, Wuhan 430060, China

Corresponding author:

**Xu-Feng Guo**,

Department of Cancer Center,

Renmin Hospital of Wuhan University, Wuhan, China,

Wuhan University at Jiefang Road 238, Wuhan 430060, RP China

Tel.: +86 27 88073385; Fax: +86 27 88042292. E-mail: [guoxufeng007@126.com](mailto:guoxufeng007@126.com)


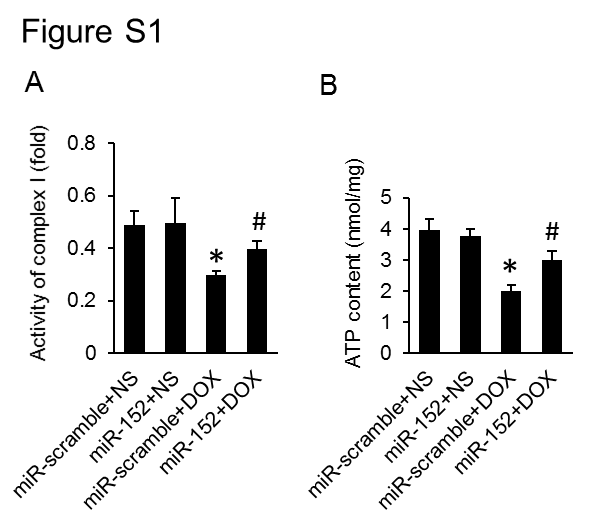


Figure S1 The alterations in mitochondrial respiration complex I activity and ATP levels. (A) mitochondrial respiration complex I activity (n=6). (B) ATP levels (n=6). Values represent the mean ± SEM. **P* <0.05 versus NS+miR-scramble, #*P*< 0.05 versus DOX+miR-scramble.
